# *Bacillus siamensis* Reduces Cadmium Accumulation and Improves Growth and Antioxidant Defense System in Two Wheat (*Triticum aestivum* L.) Varieties

**DOI:** 10.3390/plants9070878

**Published:** 2020-07-11

**Authors:** Samrah Afzal Awan, Noshin Ilyas, Imran Khan, Muhammad Ali Raza, Abd Ur Rehman, Muhammad Rizwan, Anshu Rastogi, Rezwan Tariq, Marian Brestic

**Affiliations:** 1 Department of Grassland Science, Animal Science and Technology College, Sichuan Agricultural University, Chengdu 611130, China; Muskanawan62@gmail.com (S.A.A.); Imran.62k@gmail.com (I.K.); 2 Department of Botany, Arid Agriculture University, Rawalpindi 46000, Pakistan; 2018602004@stu.sicau.edu.cn (N.I.); ibramkhan00@gmail.com (A.U.R.); 3 College of Agronomy, Sichuan Agricultural University, Chengdu 611130, China; razaali0784@yahoo.com; 4 Department of Environmental Sciences and Engineering, Government College University, Allama Iqbal Road, Faisalabad 8000, Pakistan; mrazi1532@yahoo.com; 5 Laboratory of Bioclimatology, Department of Ecology and Environmental Protection, Poznan University of Life Sciences, Piatkowska 94, 60-649 Poznan, Poland; anshu.rastogi@up.poznan.pl; 6 Jamia Masjid Sulemani, Toba Tek Singh, Punjab 36050, Pakistan; Rj.rezwan@gmail.com; 7 Department of Plant Physiology, Slovak University of Agriculture, A. Hlinku 2, 949 76 Nitra, Slovakia

**Keywords:** cadmium, wheat, antioxidants, growth, biomass

## Abstract

Bioavailability of cadmium (Cd) metal in the soils due to the scarcity of good quality water and industrial waste could be the major limiting factor for the growth and yield of crops. Therefore, there is a need for a prompt solution to the Cd toxicity, to fulfill increasing food demand resulting from growing world population. Today, a variable range of plant growth promoting rhizobacteria (PGPR) is being used at a large scale in agriculture, to reduce the risk of abiotic stresses on plants and increase crop productivity. The objective of this study was to evaluate the efficacy of *Bacillus siamensis* in relieving the Cd induced damage in two wheat varieties (i.e., NARC-2009 and NARC-2011) grown in Cd spiked soil at different concentrations (0, 20, 30, 50 mg/kg). The plants under Cd stress accumulated more Cd in the roots and shoots, resulting in severe oxidative stress, evident by an increase in malondialdehyde (MDA) content. Moreover, a decrease in cell osmotic status, and alteration in antioxidant enzymes such as superoxide dismutase (SOD), catalase (CAT), and ascorbate peroxidase (APX) were also observed in wheat plants under Cd stress. As a result, the Cd exposed plants showed a reduction in growth, tissue biomass, photosynthetic pigments, membrane stability, total soluble sugars, and amino acids, in comparison to control plants. The extent of damage was observed to be higher with an increase in Cd concentration. However, the inoculation of wheat with *B. siamensis* improved plant growth, reduced oxidative stress, and enhanced the activities of antioxidant enzymes in both wheat varieties. *B. siamensis* amendment brought a considerable improvement in every parameter determined with respect to Cd stress. The response of both wheat varieties on exposure to *B. siamensis* was positively enhanced, whereas NARC-2009 accumulated less Cd compared to NARC-2011, which indicated a higher tolerance to Cd stress mediated by *B. siamensis* inoculation. Overall, the *B. siamensis* reduced the Cd toxicity in wheat plants through the augmentation of the antioxidant defense system and sugars production.

## 1. Introduction

Agriculture is considered the big source of economy and the basic livelihoods of people in several countries of the world [1]. Agriculture as a pillar in the food industry is expected to provide food for the world’s increasing population [2]. The global agricultural regions, including cereals and especially wheat, are facing a wide spectrum of challenges, such as biotic and abiotic stresses under normal conditions [3]. Among different types of environmental stresses, heavy metal stress is getting more attention and has become a serious environmental issue in the last few decades [4]. The cadmium (Cd) is thought to be a very toxic entity, non-biodegradable, bio-accumulative, and a major wheat yield-limiting factor [5]. Cd enters the environment via geogenic and anthropogenic sources, such as fertilizer, sewage slough dispersal, industrial waste, electroplating, and atmospheric deposition [6]. The Cd firstly accumulates by roots directly from the soil and causes a significant reduction in root length, from roots it gets transfer to aerial parts and reduces the photosynthesis, resulting in a decrease of plant growth and yield [7]. Moreover, Cd leads to the excessive production of ROS responsible for oxidative damage, which negatively affects the antioxidant defense system of plants [4].

Cd has high mobility and bioavailability. It enters the food chain via the consumption of different vegetables, cereals, and cereal grains obtained from Cd contaminated soil [8]. The wheat (*Triticum aestivum* L.) is utilized as a staple food by more than 50% population of the world and an important cereal crop worldwide [2]. In recent times, the demand for wheat to feed the increasing population is getting the big attention of researchers and policymakers [9]. Wheat has a greater potential to accumulate Cd in its various parts such as root, leaves, and grains, as compared to other cereals, resulting in the higher Cd compartmentalization in wheat [10]. However, the accumulation of Cd varies with the wheat cultivars, type of soil, and soil contamination level. However, the uptake and transfer of Cd from root to shoot depend upon the xylem and phloem loading [11]. Therefore, it is imperative to reduce the intake and transfer of Cd to aerial parts, an ultimate risk to humans and other living organisms that consume wheat [12].

Plant growth-promoting rhizobacteria (PGPR) increase the root development resulting in the accumulation of water and essential nutrients to a suitable concentration and, consequently, improve the plant growth by enhancing the photosynthetic apparatus efficiency, linked with chlorophyll concentration and PSII functionality [13]. PGPR are currently used to immobilize and resist the metal toxicity and improve the plant growth by reducing the heavy metal uptake and accumulation in different parts of the plants [14]. PGPR increase the plant growth by restricting the heavy metal accumulation in roots and blocking its transfer toward aerial parts through the shoots [15]. The higher uptake of heavy metals negatively impacted the photosynthetic carbon consumption during respiration by altering the mitochondrial and electron transport chain configuration. However, inoculation with PGPR recovered the plant metabolism by limited translocation of metals in the roots of plants [13]. Previously, *Bacillus megaterium* was reported to limit the intake and transfer of nickel and improved the growth by stimulating the defense system of *Sorghum halepense* (L.)*, Luffa cylindrica* (L.) and *Brassica juncea* (L.) Czern. [16]. *Neorhizobium huautlense* was identified to considerably increase the growth and biomass production of Chinese cabbage and radish by reducing the uptake of Cd and lead (Pb) [17]. Enterobacter species have ameliorated the growth of rice seedlings concerning germination potential, biomass and chlorophyll contents by reducing the Cd stress in vitro [18]. Moreover, PGPR provide better resistance to heavy metal infected sites in plants by the synthesis of plant hormones such as indol acetic acid (IAA) and gibberellins. These also facilitate the production of siderophores and solubilizing phosphates that increase the plant growth and physiological profile by minimizing the translocation of heavy metals within the plants [19]. 

Thus, the harmful impact of heavy metals, especially Cd in crops, while stimulating the plant growth needs to be reduced. The present study explored the beneficial role of seed inoculation with *Bacillus siamensis* strain in two wheat varieties, with respect to their growth, photosynthetic pigments, oxidative damage, metal uptake, and defense system in Cd contaminated soil. This study reveals the active role of *B. siamensis* to reduce Cd-induced toxicity in wheat plants, which may provide new strategies to increase cereal crop production by ameliorating heavy metal toxicity in plants with application of PGPR.

## 2. Materials and Methods

### 2.1. Inoculation of Microbial Strains

Selected microbial strain (*Bacillus siamensis* strain no. MH559649) was provided by the Department of Botany, Arid Agriculture University, Rawalpindi, Pakistan. The strain was cultured by the addition of bacterial strains in 50 mL of nutrient broth (13 g L^−1^) medium. The flasks containing broth were placed in an incubator at 28 °C for 48 h, to let them proliferate. After the growth, the pure culture was maintained for future use. This pure culture was used to prepare the *B. siamensis* culture with an adjusted concentration of 1.2 × 10^8^ cells/ml for experimental purposes, according to the protocol of [20]. 

### 2.2. Experimental Conditions

A pot experiment was performed in the greenhouse at the Department of Botany, Arid Agriculture University, Rawalpindi, Pakistan, under natural conditions at 28/20 °C day and night temperature with 65 ± 6% relative humidity. The seeds of two wheat (*Triticum aestivum* L.) varieties (NARC-2009 and NARC-2011) were obtained from the National Agriculture Research Centre (NARC), Islamabad, Pakistan. The seeds were surface sterilized with sodium hypochloride (2.6% active chloride), then properly washed with double distilled water. Afterwards, half of the seeds of each wheat variety were inoculated with *B. siamensis* for 24 h at room temperature. Then, the inoculated seeds were air-dried and sown in the half pots, and the rest of the half pots had un-inoculated seeds (pre-treated with distilled water over night). Eight seeds of each wheat variety were sown in each plastic pot containing 5 kg air-dried loamy soil (1:3) of sand and silt, respectively. A detailed analysis of soil is provided in Table 1. Before sowing the seeds, soil was treated with the different concentration of Cd (CdCl_2_.2H_2_O), i.e., 0, 20, 30, and 50 mg/kg soil, and the remaining four treatments were given as Cd 0, 20, 30, and 50 mg/kg soil + PGPR (*B. siamensis*). The field capacity was maintained at 70% throughout the experiment. After ten days of sowing, wheat plants of each pot were subjected to thinning, and five wheat plants were kept in each pot. The experiment was carried out with three replicates of each treatment in a completely randomized design (CRD). The plants were collected for further analysis at 35 days after sowing (DAS).

### 2.3. Growth Parameters

At 35 DAS, the plants were harvested and immediately the lengths of root, shoot, leaf area, and fresh weight were measured using meter rod and electrical balance. These parameters were recorded for each pot, and the mean values were determined in triplicate. Afterward, the plant roots were washed with distilled water to remove the contamination, and then oven-dried at 70 °C for 48 h, to measure the constant dry weights and weighed [21]. 

### 2.4. Measurement of Photosynthetic Pigments

The fresh leaf samples were extracted with 85% *v/v* acetone at 4 °C for 24 h under dark conditions. Afterward, the ready sample’s wavelength was measured at 470, 647, and 664 nm by using a spectrophotometer (U-2900 Hitachi. High Technologies, Schaumburg, IL, USA). The chlorophyll contents were calculated according to the method described by [22].

### 2.5. Determination of Soluble Sugars and Water Status of Wheat 

The soluble sugars in the leaves were estimated by following the protocol of [23]. To calculate the osmotic potential and water potential the method of [24] was followed. The osmotic potential was calculated by the equation [25]. The leaf relative water content (LRWC) was calculated using the methods of [26].

### 2.6. Measurement of MDA, MSI and Antioxidant Enzyme Activities 

Malondialdehyde (MDA) content represents the lipid peroxidation was estimated by following the method of [27]. The MDA content was recorded by following the procedure of [28]. The membrane stability index (MSI) was calculated according to methods given by [29].

For the analysis of antioxidant enzymes activities, a crude enzyme extract was prepared by homogenizing 0.5 g of fresh leaf samples, replicated thrice per treatment in an extraction buffer 5 mL comparised of pre-cooled ten mM potassium phosphate buffer (pH 7.6) containing EDTA (1 mM), and 4% (*w/v*) polyvinylpyrrolidone (PVP) using a chilled pestle and mortar at 4 °C for 10 min. Afterwards, the homogenized mixture was centrifuged at 12,000× *g* for 15 minutes at 4 °C, and the supernatant was collected to use as enzyme extract for further analysis. 

Superoxide dismutase (SOD) activity was measured based on its ability to inhibit nitro blue tetrazolium (NBT) photoreduction according to the method described by [30]. The catalase (CAT) activity was assessed by following the procedure described by [31] and expressed in (U g^−1^ protein). The activity of ascorbate peroxidase (APX) was examined according to [32]. Both SOD and APX are also presented in a unit of U g^−1^ protein. 

### 2.7. Cd Uptake in Roots and Shoots and Tolerance Indexes 

The samples of roots and shoots were digested with (hydrochloric acid/nitric acid = 1:3 (*v/v*) by placing solution for 24 h at room temperature, and later, the digestion was accomplished by heating the solution on hot plate, according to standard protocols of [33]. The plant samples were oven dried at 70 °C, until a constant weight was reached. All the samples were fine grounded and transferred to conical flasks (1 g per sample) and added 5 mL of each concentrated hydrochloric acid and nitric acid (1:3). These samples were placed at room temperature overnight. The next day, nitric acid 5 (mL) was added to the mixture again and digested on hot plate until the solution was clear. After that, the solution was allowed to cool and made into a final volume of 25 mL by adding distilled water. The concentrations of cadmium in the roots and shoots were recorded by the atomic absorption spectrophotometer (Perkin Elmer A. Analyst 200, Rodgau, Germany).

In addition, the root and shoot index tolerance [34] were calculated by given formula:

$$Tolerance\;Index=\frac{Mean\;length\;in\;Cd\;solution/soil}{Mean\;length\;in\;control}$$

## 3. Statistical Analysis

The analysis of data was accomplished by using SPSS. The significance of data was analyzed with one-way analysis of variance (ANOVA). All values are given as a mean of three replicates ± standard deviation (SD). The 5% level of probability was used to compare the mean with the least significance difference (LSD) test.

## 4. Results 

### 4.1. Plant Growth, Biomass, and Photosynthesis 

**Figure 1:**
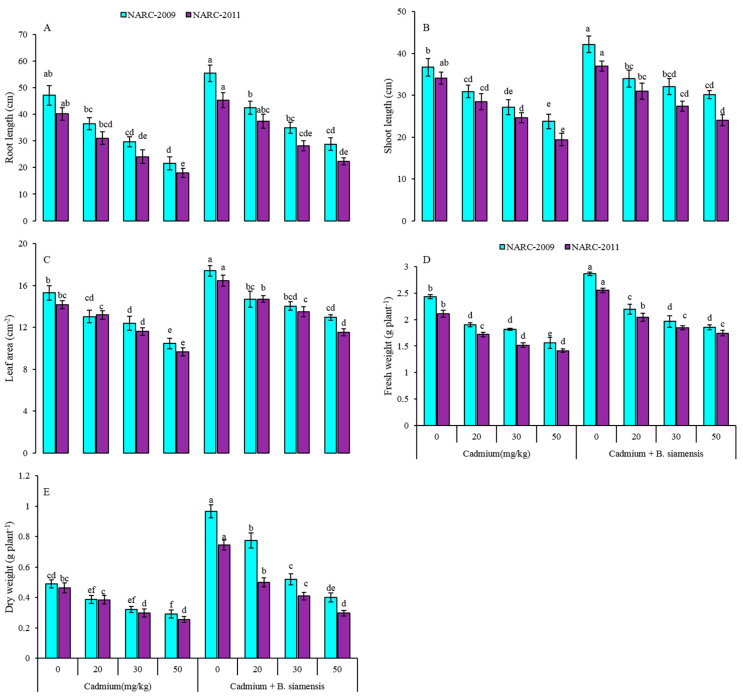
Effect of seed inoculation with *Bacillus siamensis* on root length (**A**), shoot length (**B**), leaf area (**C**), fresh weight (**D**), and dry weight (**E**) of two wheat varieties under Cd stress. Data are expressed as the means of three replicates. Bars show ± SD and different letters show a significant difference among treatments at 0.05 level.

The results of the present study depicted that inoculation of *B. siamensis* positively enhanced the growth and biomass of both wheat varieties grown in Cd-contaminated soil (Figure 1). The lengths and weights of roots and shoots, and leaf area, were recorded in both varieties. However, the Cd-stress at all tested concentrations (20, 30, and 50 mg/kg) decreased the growth and biomass of wheat whereas, maximum reduction was observed with Cd-50 mg/kg. The Cd at 50 mg/kg decreased the root length by 39% and 45%, shoot length by 18% and 30%, leaf area by 15% and 19%, fresh weight by 23% and 18%, and dry weight of plant by 18% and 35% in NARC-2009 and NARC-2011, respectively, over the respective controls (0 mg/kg Cd). The inoculation of *B. siamensis* considerably increased the length of root by 15%, shoot by 13%, leaf area by 12%, fresh weight by 15%, and dry weight of plant by 49% in NARC-2009, however, the increase in roots length by 11%, shoot lengths by 8%, leaf area by 14%, fresh weight by 17%, and dry weight of plant by 38% in NARC-2011 was noticed over the respective controls. Seed inoculation with *B. siamensis* improved the growth-related traits in both wheat varieties under Cd stress. However, the maximum reduction in growth and biomass of wheat was observed with Cd at 50 mg/kg in NARC-2011 than NARC-2009 without *B. siamensis*. 

**Figure 2:**
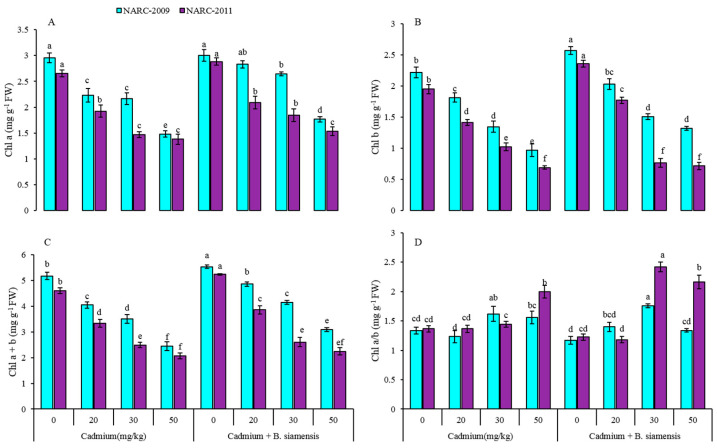
Effect of seed inoculation with *Bacillus siamensis* on chlorophyll a (**A**), chlorophyll b (**B**), chlorophyll a + b (**C**), and chlorophyll a/b (**D**) of two wheat varieties under Cd stress. Data are expressed as the means of three replicates. Bars show ± SD and different letters show a significant difference among treatments at 0.05 level.

In the current study, the inoculation of *B. siamensis* significantly affected the photosynthetic pigments, such as chlorophyll a, chlorophyll b, chlorophyll a + b, and a/b under Cd-contaminated soil (Figure 2). The decreasing trend of above parameters were observed to be greater with an increase in Cd concentration, whereas *B. siamensis* positively increased the photosynthetic pigments in both wheat varieties. The lowest values of these parameters were obtained in Cd-50 mg/kg, however, the highest values were observed at 0 mg/kg Cd with *B. siamensis*. There were increases in chlorophyll a by 2% and 8%, chlorophyll b by 14% and 17%, and total chlorophyll by 7% and 12% in NARC-2009 and NARC-2011 with *B. siamensis*, respectively, over the controls. Moreover, the maximum reduction was observed in chlorophyll a by 40% and chlorophyll b by 41% in NARC-2009 and chlorophyll a by 42% and chlorophyll b by 63% in NARC-2011 under Cd-50 mg/kg when compared with the respective controls.

### 4.2. Total Soluble Sugars and Water Status

**Figure 3:**
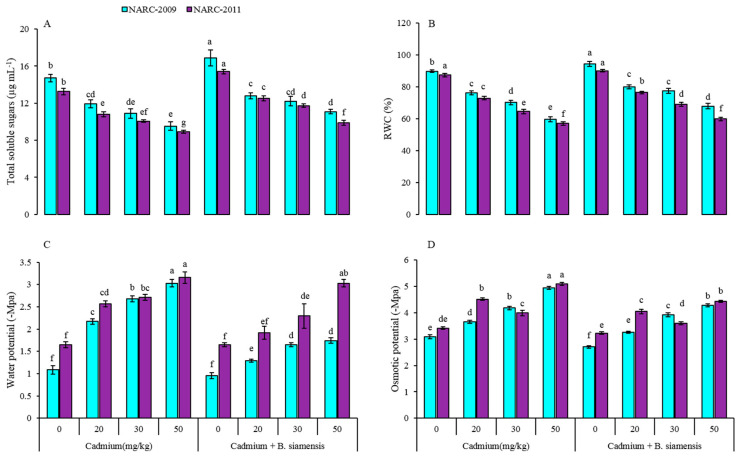
Effect of seed inoculation with *Bacillus siamensis* on total soluble sugars (**A**), relative water content (RWC) (**B**), water potential (**C**), and osmotic potential (**D**) of two wheat varieties under Cd stress. Data are expressed as the means of three replicates. Bars show ± SD and different letters show a significant difference among treatments at 0.05 level.

The results showed that total soluble sugars (TSS) were considerably improved in wheat plants with *B. siamensis* however, Cd-stress at all levels diminished these contents (Figure 3). Moreover, the Cd stress decreased the TSS linearly as from lower to higher concentration of Cd, while a higher concentration of Cd had a severe impact on both wheat varieties. The Cd at 50 mg/kg decreased the TSS by 24% and 26% in NARC-2009 and NARC-2011, as compared to the respective controls. In contrast, the inoculation of *B. siamensis* increased TSS by 13% in NARC-2009 and by 14% in NARC-2011, over the respective controls. In addition, the NARC-2009 showed less decrease in TSS than NARC-2011 under Cd-stress. 

Moreover, the water potential, osmotic potential, and relative water content (RWC) were greatly improved in wheat with *B. siamensis* over Cd- stressed plants (Figure 3). Cd-stress negatively impacted these parameters and the values of water potential, osmotic potential, and RWC were 1.74 megapascal (−Mpa), 4.27 megapascal (−Mpa), and 68% under Cd-stress at 50 mg/kg in NARC-2009, while, 3.03 megapascal (−Mpa), 4.43 megapascal (−Mpa), and 60% in NARC-2011. On the contrary, the values for water potential 0.95 megapascal (−Mpa), 1.64 megapascal (−Mpa), osmotic potential 2.7 −MPa, 3.2 −Mpa, and RWC 94%, 90% were found in NARC-2009 and NARC-2011 with *B. siamensis,* respectively.

### 4.3. MDA, MSI, SOD, CAT and APX Activities in Laves

**Figure 4:**
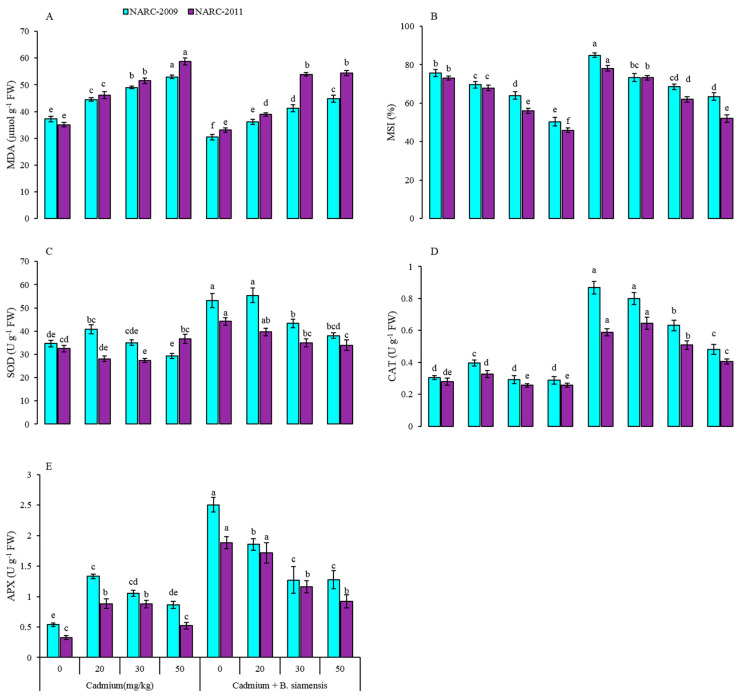
Effect of seed inoculation with *Bacillus siamensis* on malondialdehyde (MDA) (**A**), membrane stability index (MSI) (**B**), superoxide dismutase (SOD) (**C**), catalase (CAT) (**D**), and ascorbate peroxidase (APX) (**E**) of two wheat varieties under Cd stress. Data are expressed as the means of three replicates. Bars show ± SD and different letters show a significant difference among treatments at 0.05 level.

The Cd at all tested levels considerably affected the wheat plants and impact was severe at a higher Cd concentration. Higher MDA content and lower MSI were observed at 50 mg/kg Cd, which were improved by the application of *B. siamensis* as shown in Figure 4. The MDA content increased by 42% and 67% in NARC-2009 and NARC-2011 at 50 mg/kg Cd over the respective controls. However, the *B. siamensis* decreased the MDA by 22% and 6% in NARC-2009 and NARC-2011, respectively, when compared with controls. Therefore, MSI was decreased with increase in Cd stress, and a 16% and 29% decrease in MSI was found in NARC-2009 and NARC-2011 at 50 mg/kg Cd over the respective controls. In addition, the application of PGPR improved the MSI by 11% in NARC-2009 and 7% in NARC-2011, as compared to their respective controls. The response of NARC-2009 with respect to MDA and MSI was found to be improved with or without PGPR inoculation, as compared to NARC-2011. 

The inoculation of *B. siamensis* positively impacted the SOD, CAT, and APX activities in wheat under Cd-contaminated soil (Figure 4). The SOD, CAT, and APX activities diminished in leaves with Cd stress, and were decreased with increasing Cd levels. The Cd at 50 mg/kg decreased the SOD activity by 10%, 5%, CAT by 57%, 46%, and APX by 137%, 182% in NARC-2009 and NARC-2011 over the respective controls. On the other hand, the *B. siamensis* increased the SOD, CAT, and APX activities by 35%, 65%, and 78% in NARC-2009 and, 27%, 53%, and 83% in NARC-2011 with respective controls. The activities of these antioxidant enzymes were found maximum at Cd 0 mg/kg with *B. siamensis* in both wheat varieties, as shown in Figure 4. 

### 4.4. Accumulation of Cd and Tolerance Indexes of Roots and Shoots

**Figure 5:**
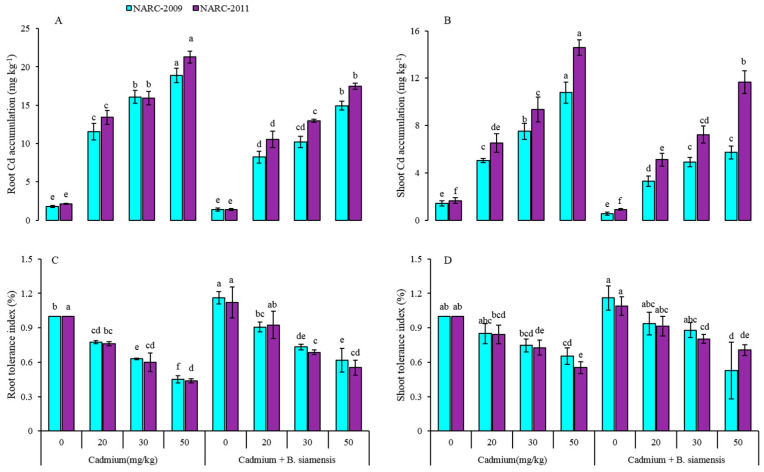
Effect of seed inoculation with *Bacillus siamensis* on Cd accumulation in root (**A**), shoot (**B**), root tolerance index (**C**), and shoot tolerance index (**D**) of two wheat varieties under Cd stress. Data are expressed as the means of three replicates. Bars show ± SD and different letters show a significant difference among treatments at 0.05 level.

The accumulation of Cd was observed in both wheat varieties, and *B. siamensis* noticeably impacted the accumulation of Cd in wheat. An obvious enhancement in Cd accumulation was noticed in both wheat varieties under all Cd concentrations, however, *B. siamensis* significantly reduced the Cd uptake in wheat shoots and roots (Figure 5). There was an 8-fold increase in Cd uptake in the roots and 6.5-fold in the shoots of NARC-2009, and by 9-fold in the roots and 7.8-fold in the shoots of NARC-2011 at 50 mg/kg Cd. Whereas the *B. siamensis* with 0 mg/kg Cd decreased the Cd accumulation by 25% and 21% in the roots, and 88%, 25% in the shoots of NARC-2009 and NARC-2011, with respect to Cd-50 mg/kg. In addition, the roots and shoots tolerance indexes were found improved in NARC-2009, as compared with NARC-2011 on exposure to the PGPR application, as shown in (Figure 5C,D).

## 5. Discussion

In the current study, two wheat varieties were grown in Cd-contaminated soil prior to the treatment with or without PGPR (*B. siamensis*). The results depicted a significant decrease in plant growth under Cd stress without amendment of *B. siamensis* (Figure 1). The Cd-toxicity related observations are similar to previous studies on other plant species [20,21,35]. However, the detailed molecular mechanism of Cd toxicity is poorly understood; however, previously researchers have explained the damaging effects that Cd may destroy the soil microbial communities, reduce the water and nutrients uptake, and impair the cell division and elongation process, which ultimately decrease crop growth and yield [20]. The decrease in the growth of wheat might be due to the damaging impact of Cd on plants morpho-physiological and biochemical attributes, and mineral uptake by plants [36]. Another possible reason is the decline in root and shoot growth due to Cd stress, which is directly associated with the inhibition of root and shoot metabolism, and ultimately affects the overall plant growth [20]. In our study, we depicted the beneficial role of seed inoculation with *B. siamensis* on plant growth and development. Some pieces of evidence highlighted the efficient role of microbes on the broad spectrum in the improvement of plant growth under stressful conditions [37]. PGPR can increase plant growth and biomass by decreasing the uptake and accumulation of metals in plants via altering the metal species in the soil [38]. Microorganisms facilitate plant growth and development and increase the supply of phosphate through siderophores formation, root hair growth, and hormonal stimulation, which reduce the heavy metal translocation [19]. The PGPR induce changes in metabolic activities involved in the solubilization and mineralization of organic phosphorous. These metabolic activities help in the efflux of proton and other various anions, and then phosphatase enzymes release that enables the hydrolysis and mineralization of phosphorus [39]. Therefore, *Bacillus* species have more capability to induce physiological changes in crop plants by secreting various metabolites that improve plant growth under unfavorable conditions [40].

Cd reduced the surface area of cells that absorb water, indicating the disturbance of the water balance [41]. However, the PGPR improves the LRWC and water potential in different plant species exposed to different types of environmental stresses [42]. It is reported that PGPR improves the stomatal aperture to uptake more water via roots and enhances the stomatal conductance, as compared to non-PGPR inoculated plants [43]. PGPR enhanced the water uptake, RWC, and membrane stability in the leaf of maize plant under Cd stress which supports our study [35]. Moreover, PGPR efficiently improved the tolerance ability of plants exposed to various environmental stresses, including heavy metals, and increased the yield of plants [44].

Chlorophyll, as a major component of the chloroplast, is efficiently associated with plant photosynthetic ability; whereas Cd and rest of the heavy metals negatively affected the chlorophylls and caused chlorosis in leaves [3]. Several types of stresses especially heavy metals, such as Cd, Zn, Cu, Hg, and Pb, induce toxicity in the cell wall and thylakoid membrane integrity, which leads to the inhibition of enzymes, i.e., Rubisco, chlorophyll synthase, involved in the synthesis of chlorophyll and resulting in the degradation of chlorophylls [45]. The decrease in photosynthetic pigments indicated the severe oxidative stress in leaves under Cd toxicity [21]. The authors of [46] observed significant damage to photosystem II with exposure to 25 µM Cd in barley landraces. The photosynthates produced by plants with the help of chlorophyll directly linked with the increase in plant biomass production, whereas a decline in chlorophylls leads to lower biomass production influenced by Cd stress [20]. The increase in nitrogen content, an important molecule of chlorophyll structure, was observed in PGPR inoculated *Medicago lupulina* L., which is associated with greater production of plant biomass under heavy metal stress [47]. Moreover, the Cd stress severely affected the membrane permeability and enhanced the protein degradation in *Brassica juncea* (L.) Czern. [48]. However, our results are in line with [18], which revealed that Enterobacter species has stimulated the growth and improved the chlorophyll content in *Oryza sativa* L. seedlings by reducing the toxicity of Cd stress. 

Heavy metal stress caused severe oxidative stress in plants thereby induces changes in the activities of antioxidant enzymes, depending upon the plant species, the growth stage of plant, dose, and duration of metals [49]. Our results depicted an increased level of MDA content, and lowered activities of antioxidant enzymes (such as SOD, CAT, and APX) and MSI in wheat under higher Cd concentration, without the amendment of *B. siamensis*. Higher Cd concentration in plants caused severe oxidative damage, due to the excessive production of ROS, which influenced the defense system of plants [3,50]. These changes critically disrupt the normal physiological mechanisms in plants, and consequently reduce the growth and yield [38]. Another study revealed that Cd induced oxidative stress, negatively affected the DNA, RNA, and ribosomal biosynthesis, and inhibited the synthesis of various enzymes necessary to reduce oxidative damage [51]. Another study revealed that Cd stress increased the MDA content which enhanced the membrane damage in the mustard plant which might lead to oxidative stress in plants [48,52]. The seed inoculation with *B. siamensis* significantly ameliorated the Cd-induced oxidative stress in wheat, as indicated by lower MDA, higher MSI, and increased activities of the antioxidant enzyme under Cd-stress. PGPR stimulated and improved the activities of the antioxidant defense system, and reduced the oxidative damage in plants under heavy metal stress [47,53]. It has been reported that the expression level of SOD, CAT, and APX was increased in plants under Cu stress after inoculation with rhizobia [54]. The SOD efficiently converts the O_2_ to H_2_O_2_ and CAT decomposes H_2_O_2_ to water and oxygen molecules. Moreover, the other antioxidant enzymes also scavenge ROS and reduce oxidative stress [21]. Some pieces of evidence showed that Cd stress increased the oxidative stress via the overproduction of ROS and enhanced MDA contents, and negatively influenced the defense system of plants [4,21,55]. The coinoculation of *Sinorhizobium* and *Agrobacterium* significantly enhanced the SOD, CAT, and APX activities in alfalfa under heavy metal stress, which might be due to the production of acidic exopolysaccharides, which act as a diffusion barrier against ROS [17,56]. It is suggested that inoculation with PGPR enhanced nitrogen and mineral nutrition and antioxidant enzyme activities, thereby reducing the uptake of heavy metals and ultimately increasing the growth of plants [47]. The lower MDA and higher activities of antioxidant enzymes in wheat indicated that *B. siamensis* reduced the oxidative stress in plants, which might be due to the reduced uptake of Cd concentrations under *B. siamensis* application over the non-treated plants. 

The seed inoculation with *B. siamensis* reduced the uptake of Cd in wheat than non-inoculated plants. PGPR significantly reduced the uptake of Cd in the root and shoot of tomato [21]. The PGPR, *Pseudomonas aeruginosa* reduced the uptake of Zn in wheat root and shoot grown under Zn metal stress [57]. Another study revealed the reduced Cd uptake in plants roots and shoots with significant increase in sorghum plant growth subjected to inoculation with microbes than non-inoculated plants, which might be due to sequestration or immobilization of Cd in the soil and, consequently, the lower availability of Cd to plants [58]. It has been reported that *B. licheniformis* colonizes the roots of *Spinacia oleracea* L., reduces the Cd uptake, and enhances the Cd stress tolerance [59]. The inoculation with *B. siamensis* decreased the Cd uptake and concentration in wheat root and shoot, which reflects that inoculation with this bacterial strain can increase the plant growth and physiological processes by reducing the Cd accumulation in wheat. However, further studies are needed in this regard with this bacterial strain. Overall, it is concluded that Cd stress severely affected the growth of both wheat varieties, while the inoculation with PGPR reduced the Cd toxicity and improved plant growth attributes. However, further studies are needed to find out the actual mechanism of Cd toxicity in plants at the molecular level, with the application of PGPR.

## 6. Conclusions

It is concluded that *B. siamensis* improved the growth and reduced the accumulation of Cd in roots and shoots of wheat. Cd stress negatively influenced the morphology, physiology, and defense system of wheat plants, however, *B. siamensis* enhanced the activities of antioxidant enzymes, such as SOD, CAT, and APX, and lowered the oxidative stress as MDA content. In contrast, the NARC-2009 performed well as compared to NARC-2011 under Cd stress, with or without PGPR application. Overall, the seed inoculation with *B. siamensis* might be an effective technique to reduce Cd and enhance growth of wheat. However, further studies are needed to explore the impact of seed inoculation with *B. siamensis* on the soil biological activities under the various experimental conditions.

## Figures and Tables

**Table 1:** Physiochemical properties of soil. Values are the means of three replicates. The values with ± show standard deviation (SD).

Physiochemical Properties	Values ± SD
pH	7.45 ± 0.002^c^
EC dSm^−1^	1.28 ± 0.003^gh^
Organic matter (%)	1.92 ± 0.006^fg^
Phosphorus (mg/kg)	6.2 ± 0.10^d^
Potassium (mg/kg)	100 ± 1.13^a^
Zn (mg/kg)	1.04 ± 0.01^gh^
Cu (mg/kg)	0.71 ± 0.008^h^
Mn (mg/kg)	2.64 ± 0.002^f^
Fe (mg/kg)	2.95 ± 0.004^f^
Cd /bioavailable Cd (mg/kg)	4.36 ± 0.002^e^/0.49 ± 0.003^i^
Saturation (%)	33 ± 0.3^b^
